# Supplementary material for: Tissue-specific modulation of CRISPR activity by miRNA-sensing guide RNAs
Source: Nucleic Acids Res. 2025 Jan 22;53(2):gkaf016. doi: 10.1093/nar/gkaf016 (PMC11754125; doi:10.1093/nar/gkaf016)
Supplement: gkaf016_Supplemental_Files [file gkaf016_supplemental_files.zip › Supplementary Figures_REVISED_CLEAN.docx]

**SUPPLEMENTARY FIGURES**


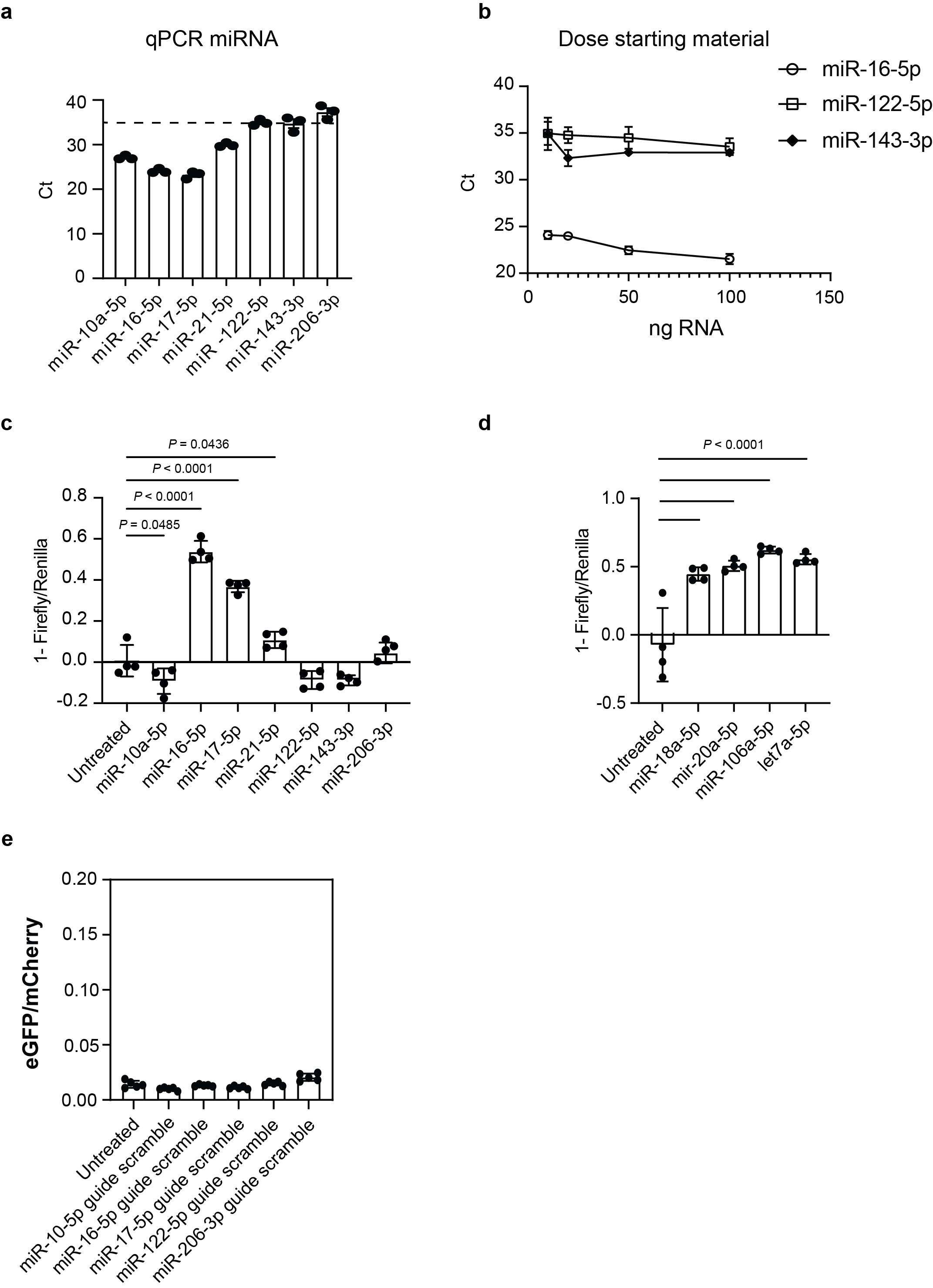


**Supplementary Fig. 1: miRNA quantification and activity. a.** Expression of miR-10-5p, miR-16-5p, miR-17-5p, miR-21-5p, miR-122-5p, miR-143-3p, and miR-206-3p in HEK293T Stoplight^+^ SpCas9^+^ assayed by qPCR. The threshold for detection of expression was set at Ct = 35 cycles (*n* = 3). **b.** Dose curve using increasing amounts of starting material (extracted RNA) as template for cDNA production (10, 20, 50 and 100 ng of RNA). Expressed miRNA miR-16-5p shows a reduction in Ct values as the dose of starting material increases, whereas the Ct value for non-expressed miRNAs (miR-122-5p and miR-143-3p) remains approximately constant (*n* = 3). **c,d.** Dual-luciferase miRNA activity data. Sensors for the miRNAs used respectively in Fig. 1e and Supplementary Fig. 6c were built and transfected into the HEK293T Stoplight^+^ SpCas9^+^. Data was collected 48 h later and presented as 1-(Firefly/Renilla luciferase luminescence, normalized to untreated) to represent miRNA activity (*n* = 4). **e.** Activities of control miR-sgRNAs with scrambled miRNA binding site sequences (cf. Fig. 1e) (*n* = 5). All data were analysed using one-way ANOVA and Dunnet’s multiple comparison test. The data represents the mean ± S.D.


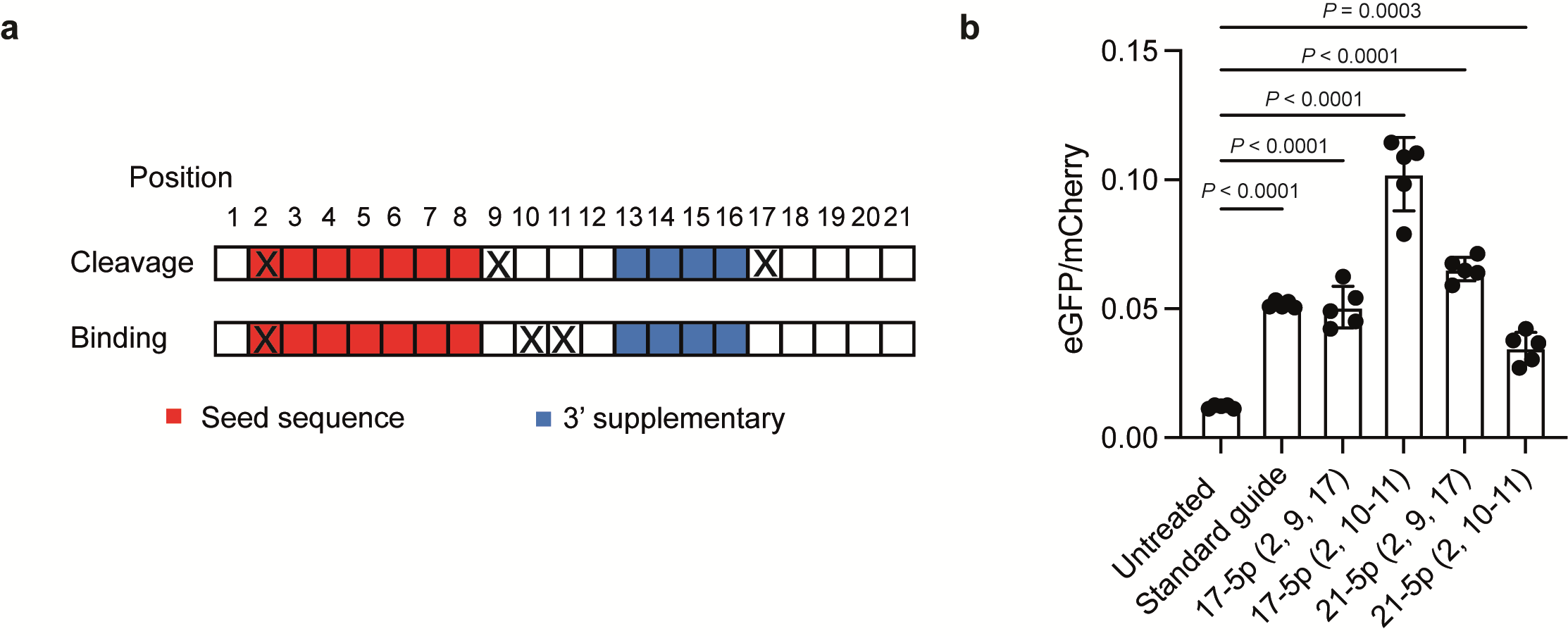


**Supplementary Fig. 2: RISC-mediated interaction modality. a.** Diagram depicting the location of the targeted mismatches based on their effect on RISC downstream modality. **b.** RISC-mediated cleavage or binding as the miR-guide activation mechanism. miR-guides responsive to miR-17-5p and miR-21-5p were made with cleavage-permissive mismatches (2, 9, and 17) and cleavage-abolishing mismatches (2, 10-11). There are no significant differences between mismatch profiles suggesting that binding alone is sufficient to cause activation (*n* = 5). All data were analysed using one-way ANOVA and Dunnet’s multiple comparison test. The data represents the mean ± S.D.


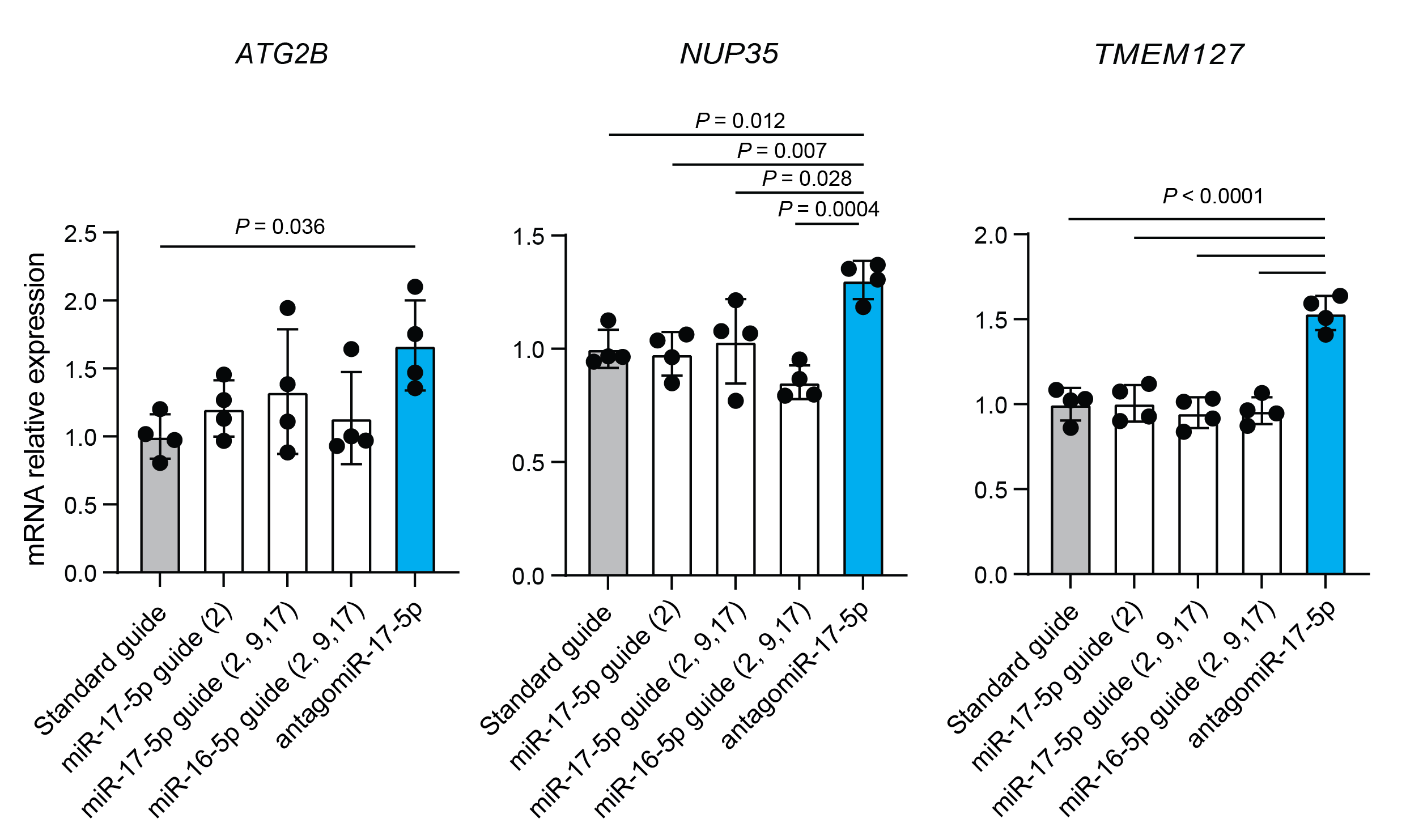


**Supplementary Fig. 3: Effect of miR-17-5p sgRNA on the expression of miR-17-5p-regulated transcripts.** Expression of *ATG2B*, *NUP35*, and *TMEM127* transcripts relative to *HPRT* housekeeping gene and normalised to a standard guide control. We tested a seed-only miR guide (miR-17-5p (2) guide), a full-miRNA miR guide (miR-17-5p (2,9,17)), and a full-miRNA miR guide responsive to a different miRNA (miR-16-5p (2, 9, 17) guide). We observed no statistically different changes between miR guides sgRNA, and the standard guide control, suggesting that the trigger hairpin does not affect mRNA homeostasis (*n* = 4). All data were analysed using one-way ANOVA and Dunnet’s multiple comparison test. The data represent the mean ± S.D. Histograms for standard guide and antagomiR treatments are coloured in grey and blue, respectively.


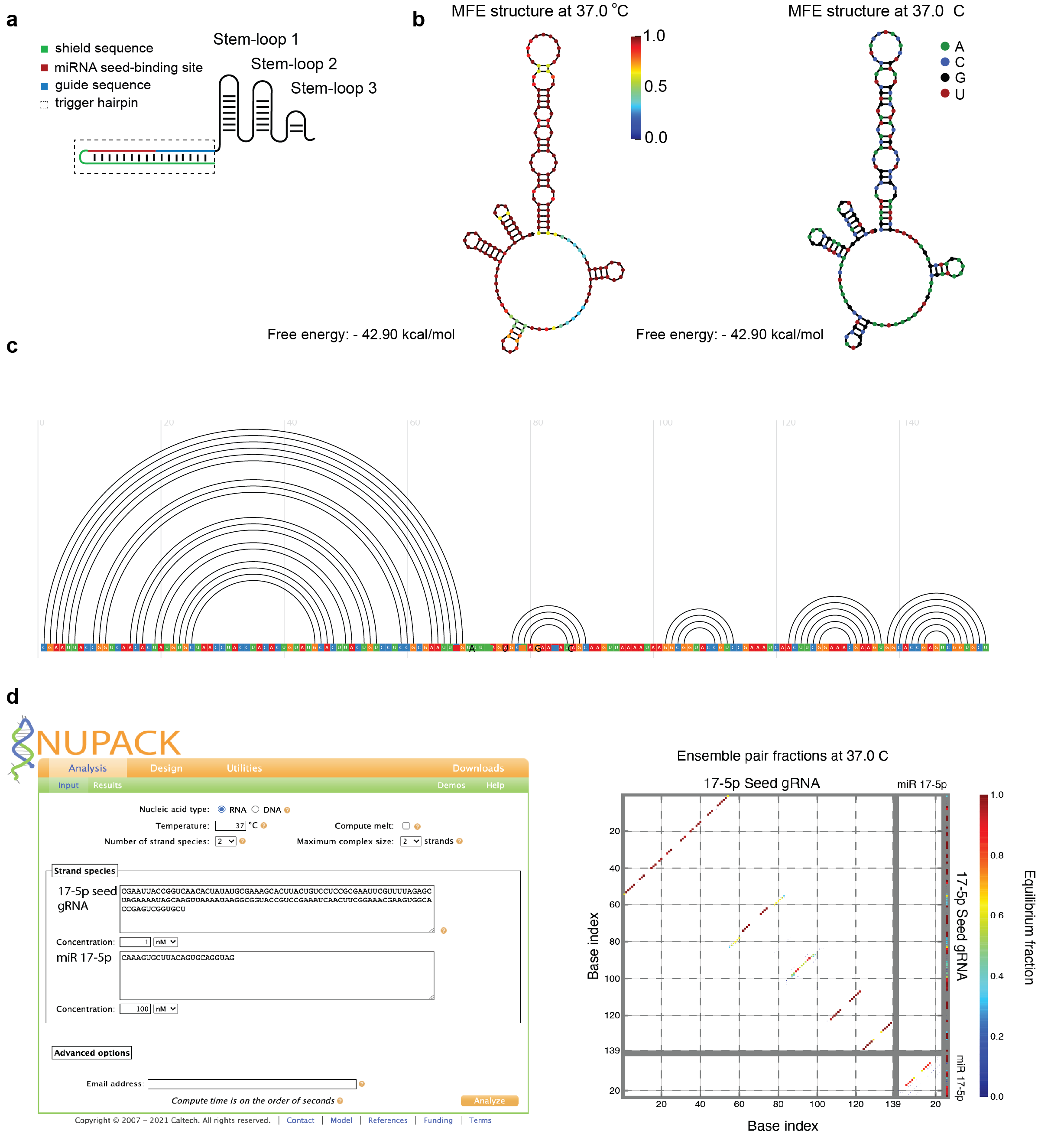


**Supplementary Fig. 4: Structure of a miR-guide. a.**
Structural features of a miR-guide. **b** Using NUPACK, we calculated the most common conformation in the equilibrium ensemble of the miR-guide. The probability that each nucleotide is found in the specified position (left) and a colour-coded depiction of the miR-guide sequence in this structure (right) are shown. **c.** CoFold calculation of the secondary structure of the same miR-17-5p miR-guide after simulated co-transcriptional folding. **d.** Equilibrium secondary structure of miR-17-5p guide (1 nM) in the presence of miRNA 17-5p (100 nM) calculated using NUPACK. Left panel shows the parameters, species, sequences and concentrations used. We chose a 100 times excess miRNA to sgRNA ratio to favour hybridization, while the concentration was set to the nM range based on typical miRNA concentrations in a mammalian cell. Right panel shows the hybridization map indicating the equilibrium probability of each possible hybridized base pair. No inter-strand interaction is predicted, suggesting that the mechanism of activation of the miR-guide is not simple strand displacement and is therefore likely to be mediated by AGO, as designed.


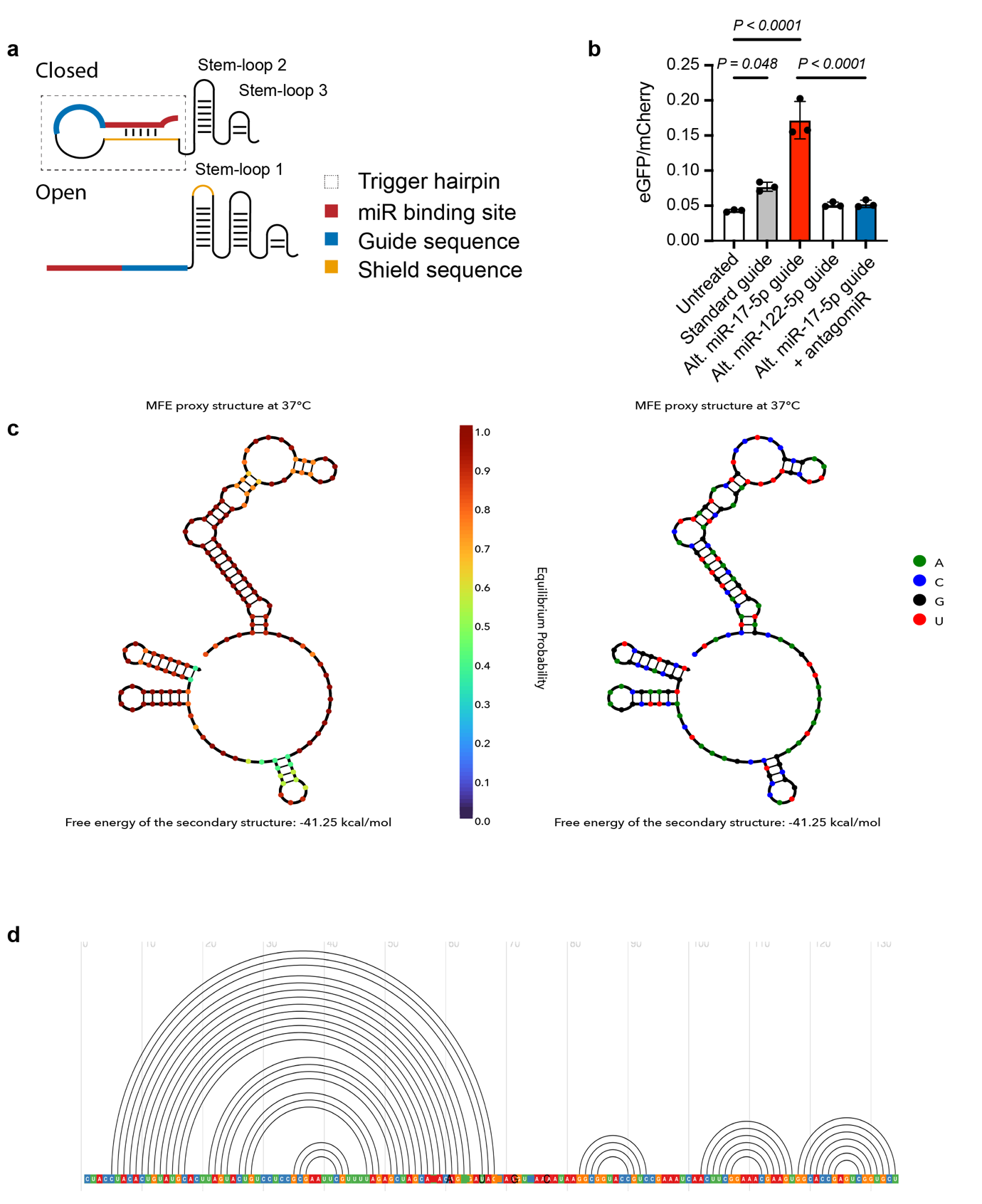


**Supplementary Fig. 5: miR-guide operating under an alternative design.** **a.**
Structural features of an alternative miR-guide. By understanding the interaction between the miR guides and AGO, we tested the impact of trigger hairpin location. **b**. Transfection of HEK293T Stoplight^+^ SpCas9^+^ using alternative miR guides. 100 ng of each plasmid condition were transfected in reverse and results were collected 72 h later. AntagomiR against miR 17-5p were co-transfected with the plasmid at a final concentration in the well of 500 nM (*n = 3)*. **c.** Calculations of the most common conformation in the equilibrium ensemble of the miR-guide using NUPACK. The secondary structure displays a gradient coloration denoting the probability that each nucleotide is found in that specified position (left). On the right, a depiction of the alternative miR-guide sequence within this structure (right) is shown. **d.** CoFold calculation of the secondary structure of the same miR-17-5p miR-guide after simulated co-transcriptional folding. Data were analysed using one-way ANOVA and Tukey’s multiple comparison test.

**
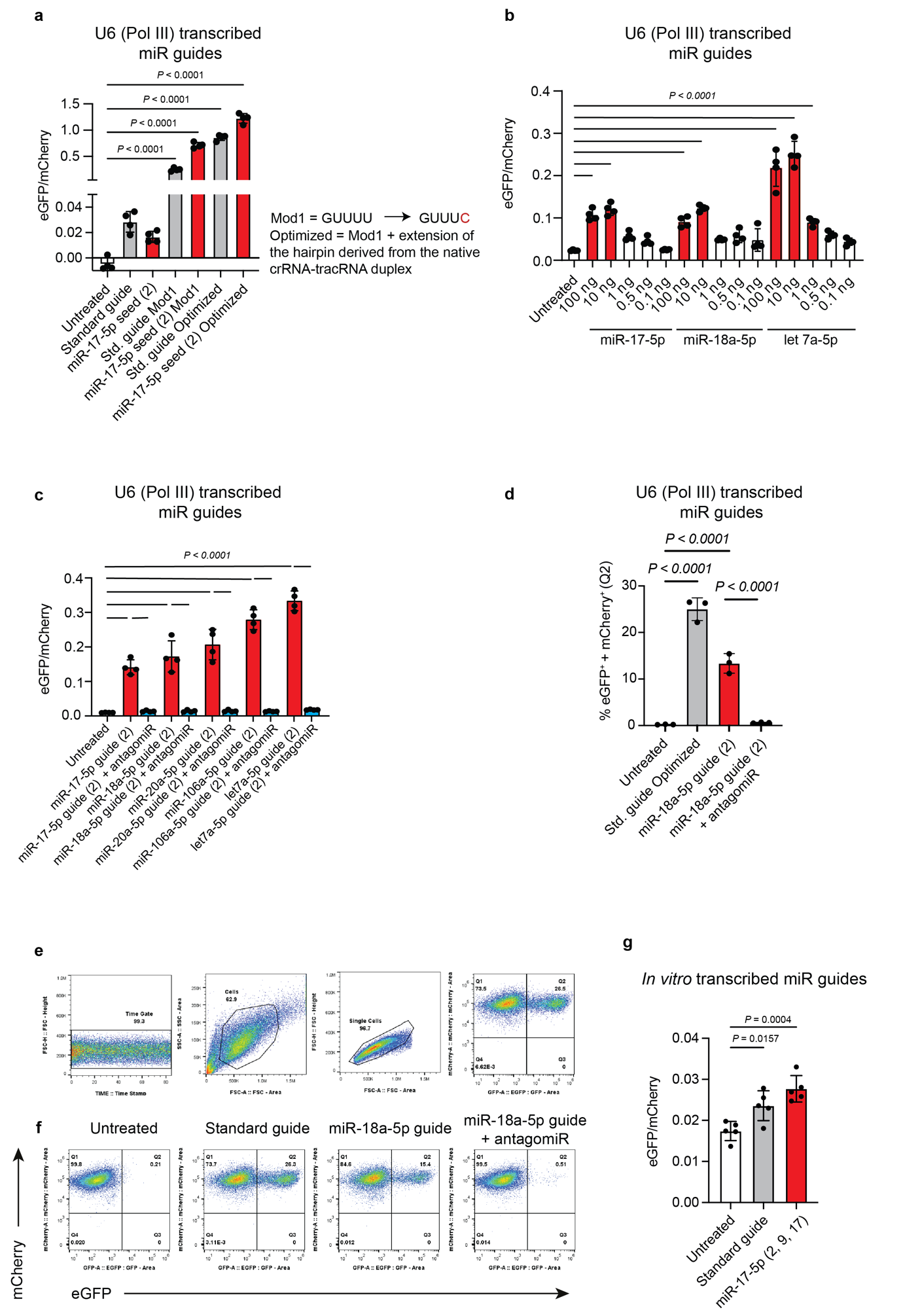
**

**Supplementary Fig. 6: sgRNA backbone optimisation increases activity.** **a.** The activities of miR-guides transcribed from a Pol III promoter increase by approx. 50× when the four consecutive Us in the repeat region are changed to three Us and one C (labelled as Mod1), reducing polymerase stalling. (Note the change in axis scaling at the break). A further known modification of the sgRNA backbone, extension of the hairpin derived from the native crRNA-tracRNA duplex, resulted in a further increase in activity to approx. 75× (labelled as Optimized). (*n* = 4). **b.** Dose-responsive effect of CRISPR MiRAGE upon decreasing doses of optimized, seed-only miR-guides (*n* = 4). **c.** Further characterization of CRISPR MiRAGE using antagomiRs against miR 17-5p, 18a-5p, 20a-5p, 106a-5p and let-7a-5p. The activity of these miRNA was assessed in supplemental figure 1c and 1d. HEK293T Stoplight^+^ SpCas9^+^ cells were transfected with 1 ng plasmid and co-transfected, where indicated, with its respective antagomiR at a final concentration of 500 nM (*n* = 4). **d.** Cytometry measurement of HEK293T Stoplight^+^ SpCas9^+^ transfected with 5 ng of plasmid and antagomir at 500 nM in a 24 well plate (*n = 3)*. **e.** Gating strategy followed: a gate on time was used to guarantee that the analysis is performed only when the measurement was stable increasing the accuracy of the analysis. Cells were identified according to their light scatter parameters (forward scatter or FSC and side scatter or SSC-A). Subsequently, a double gating was used to exclude doublets (FSC-A vs FSC-H). Expression of eGFP and mCherry was assessed using a bivariant dot plot within the Untreated, miR-18a-5p and Standard guide HEK293T Stoplight^+^ SpCas9^+^ cells. Quadrant regions (Q1 to Q4) are showing the percentage of cells in each sub-population where Q2 defines the double positive population (eGFP^+^mCherry^+^). **f.** Representative dot plot per condition (untreated, standard guide, miR-18a-5p and miR-18a-5p + antagomiR) after collecting 50,000 events. Q2 shows eGFP^+^mCherry^+^ cells. **g.** Transfection of an *in vitro* transcribed active (full-miRNA miR-17-5p guide with wildtype backbone) at a final concentration of 50 nM resulted in detectable editing activity (*n* = 4). Data were analysed using one-way ANOVA and Dunnet’s multiple comparison test, except in **c,d** which used Tukey’s multiple comparison correction. The data represent the mean ± S.D. Histograms for standard guide, significant miR-guides, and antagomir-treated samples are coloured in grey, red, and blue respectively.


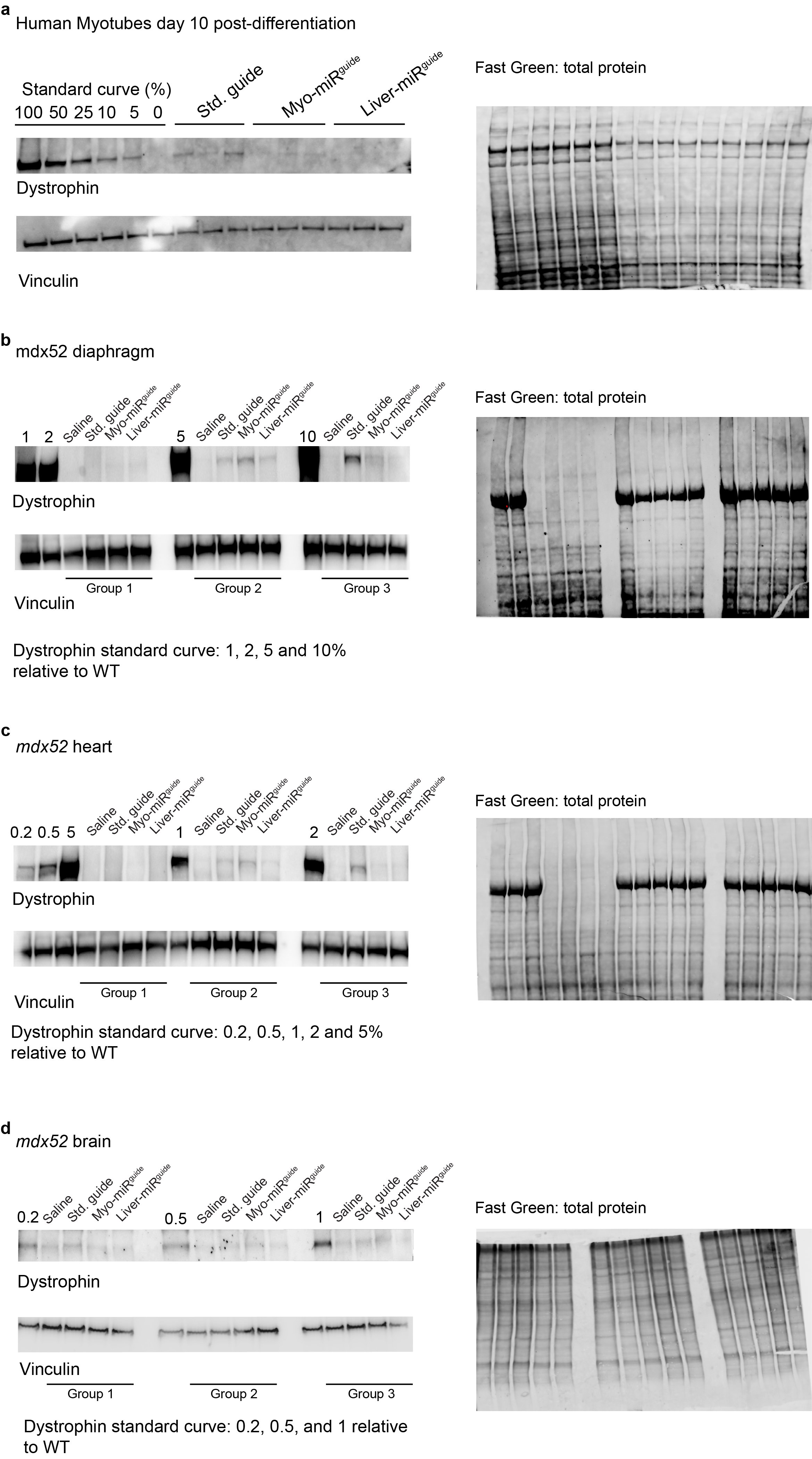


**Supplementary Fig. 7: Dystrophin restoration Western blots. a.** Western blot used for the quantification of dystrophin restoration in Δ52 human myotubes shown in Fig. 4e. A total of 15 μg of protein were blotted against dystrophin and vinculin (loading control). Total protein stain of the membrane using Fast Green FCF is shown on the right (*n* = 3). **b, c.** Western blots used for the quantification of dystrophin restoration in *mdx52* diaphragm and heart shown in **Fig. 4i**. A total of 60 μg of protein were blotted against dystrophin and vinculin (loading control). Total protein stain of each membrane using Fast Green FCF is shown on the right (*n* = 3). **d.** Western blots for quantifying the amount of dystrophin in *mdx52* brain. A representative set containing all conditions is shown in Fig. 4i. A total of 80 μg of protein were blotted against dystrophin and vinculin. Total protein stain of the western blot membrane using Fast Green FCF is shown on the right (*n =* 3).


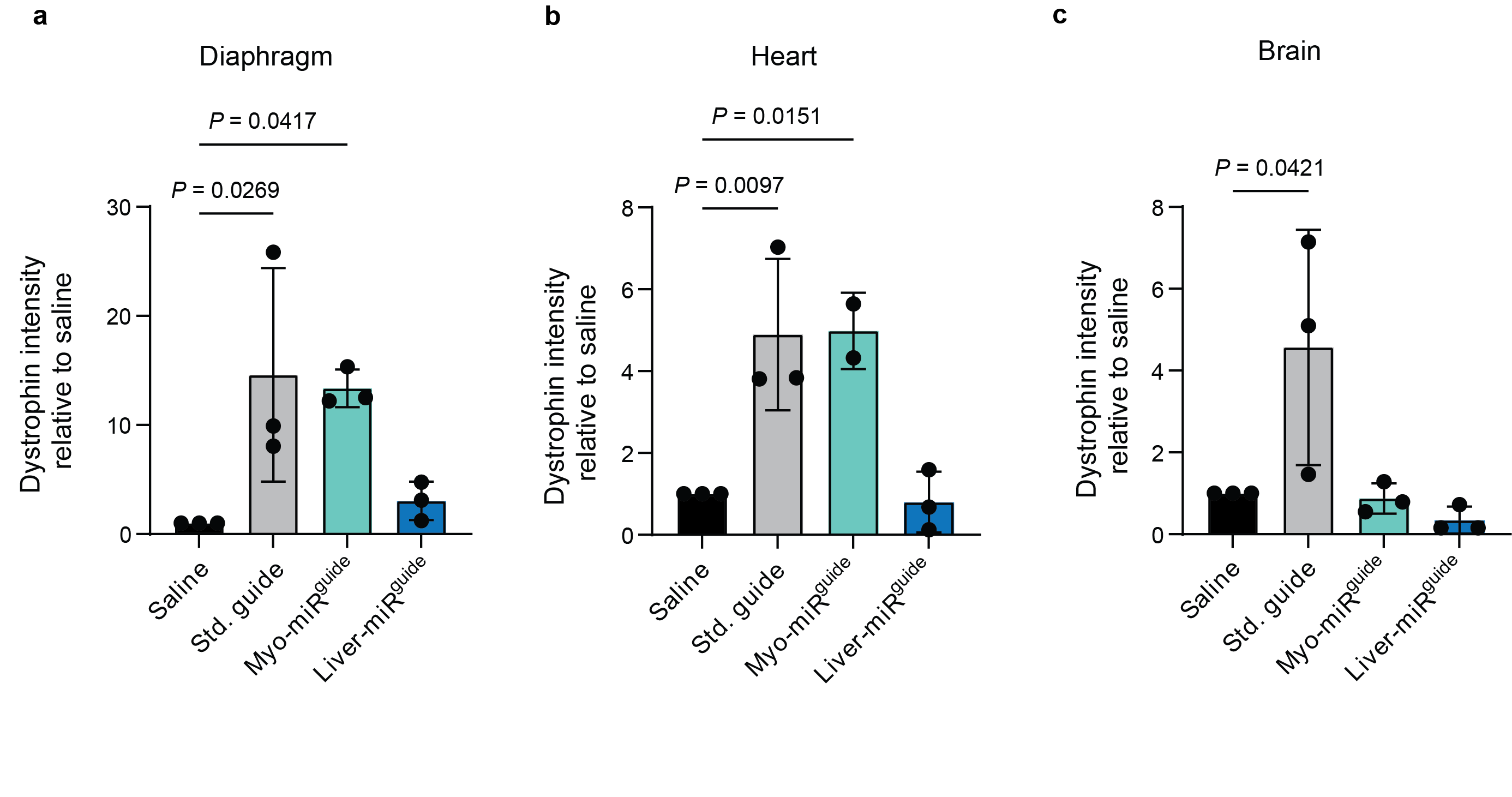


**Supplementary Fig. 8: Quantification of dystrophin restoration.** Quantification of dystrophin production from the western blots shown in Supplementary Fig. 7. Dystrophin levels are normalized to Fast Green signal for each sample and expressed as fold change to those measured for saline-treated animals (*n = 2-*3). **a.** Diaphragm. **b.** Heart. **c.** Brain. The data represent the mean and the error bars ± S.D.
