# Supplementary material for: Tissue-specific modulation of CRISPR activity by miRNA-sensing guide RNAs
Source: Nucleic Acids Res. 2025 Jan 22;53(2):gkaf016. doi: 10.1093/nar/gkaf016 (PMC11754125; doi:10.1093/nar/gkaf016)
Supplement: gkaf016_Supplemental_Files [file gkaf016_supplemental_files.zip › Supplementary Table 1.docx]

**SUPPLEMENTARY TABLE 1**

**List of sgRNA sequences used in this study**

A “standard” single guide RNA comprises a **5’ guide sequence** (that specifies the dsDNA target) contiguous with a **sgRNA backbone** comprising essential elements of the tracrRNA. A MiRAGE miR guide has additional components at the 5’ end: a **shield domain** and a **miRNA binding site**. The shield domain is partially complementary to the miRNA binding site and to the guide sequence, to which it hybridizes to form the trigger hairpin. In order to make a successful trigger hairpin, a continuous number of base pairs hybridized to the guide sequence seed region (around 6-12 bp, depending on your guide sequence) is recommended. The number of mismatches across the shield sequence should be kept to 2-5 nt, while maintaining the whole trigger hairpin free energy ~15-20 kcal/mol. This can be tuned manually via NUPACK calculations. A successful trigger hairpin should behave as a molecular zipper, where everything is kept in place by the continuous base pairs hybridized to the guide sequence‘s seed region and produce sequential opening of the trigger hairpin once they are melted. Each trigger needs to be optimized.

These essential elements are colour-coded in the table below.

| **ID** | **Sequence (5' to 3')** |
| --- | --- |
| **WT sgRNA backbone** | GTTTTAGAGCTAGAAAATAGCAAGTTAAAATAAGGCGGTACCGTCCGAAATCAACTTCGGAAACGAAGTGGCACCGAGTCGGTGCT |
| **Mod1 sgRNA backbone** | GTTTCAGAGCTAGAAAATAGCAAGTTAAAATAAGGCGGTACCGTCCGAAATCAACTTCGGAAACGAAGTGGCACCGAGTCGGTGCT |
| **Optimized backbone** | GTTTCAGAGCTATGCTGGAAACAGCATAGCAAGTTGAAATAAGGCTAGTCCGTTATCAACTTGAAAAAGTGGCACCGAGTCGGTGCT |
| **Stoplight guide sequence 17 bp** | CTGTCCTCCGCGAATTC |
| **Anti-human dystrophin guide sequence** | AAGAACACCTTCAGAACCGG |
| **Anti-mouse dystrophin guide sequence** | AATTCAGATTCAGTGºGGATG |
| **Stoplight Standard guide** | CTGTCCTCCGCGAATTCGTTTTAGAGCTAGAAAATAGCAAGTTAAAATAAGGCGGTACCGTCCGAAATCAACTTCGGAAACGAAGTGGCACCGAGTCGGTGCT |
| **Fig. 1d. 0 mismatches** | CGAATTACCGGTCAACACAATGTGCAAACCTACCTGCACTGTAAGCACTTTCTGTCCTCCGCGAATTCGTTTTAGAGCTAGAAAATAGCAAGTTAAAATAAGGCGGTACCGTCCGAAATCAACTTCGGAAACGAAGTGGCACCGAGTCGGTGCT |
| **Fig. 1d. 1 mismatches (9)** | CGAATTACCGGTCAACACAATGTGCTAACCTACCTGCACTGTATGCACTTTCTGTCCTCCGCGAATTCGTTTTAGAGCTAGAAAATAGCAAGTTAAAATAAGGCGGTACCGTCCGAAATCAACTTCGGAAACGAAGTGGCACCGAGTCGGTGCT |
| **Fig. 1d. 2 mismatches (9, 17)** | CGAATTACCGGTCAACACAATGTGCTAACCTACCTACACTGTATGCACTTTCTGTCCTCCGCGAATTCGTTTTAGAGCTAGAAAATAGCAAGTTAAAATAAGGCGGTACCGTCCGAAATCAACTTCGGAAACGAAGTGGCACCGAGTCGGTGCT |
| **Fig. 1d. 2 mismatches (2, 9)** | CGAATTACCGGTCAACACTATGTGCTAACCTACCTGCACTGTATGCACTTACTGTCCTCCGCGAATTCGTTTTAGAGCTAGAAAATAGCAAGTTAAAATAAGGCGGTACCGTCCGAAATCAACTTCGGAAACGAAGTGGCACCGAGTCGGTGCT |
| **Fig. 1d. 3 mismatches (2, 9, 17)** | CGAATTACCGGTCAACACTATGTGCTAACCTACCTACACTGTATGCACTTACTGTCCTCCGCGAATTCGTTTTAGAGCTAGAAAATAGCAAGTTAAAATAAGGCGGTACCGTCCGAAATCAACTTCGGAAACGAAGTGGCACCGAGTCGGTGCT |
| **Fig. 1e. miR-10-5p guide** | CGAATTACCGGTCAACTCTCAATGAACGTCACAAAATCGGACGAACAGGGACTGTCCTCCGCGAATTCGTTTTAGAGCTAGAAAATAGCAAGTTAAAATAAGGCGGTACCGTCCGAAATCAACTTCGGAAACGAAGTGGCACCGAGTCGGTGCT |
| **Fig. 1e. miR-16-5p guide** | CGAATTACCGGTCAACACTGATGCTAGTAGCCGCCATTATTTACATGCTGCACTGTCCTCCGCGAATTCGTTTTAGAGCTAGAAAATAGCAAGTTAAAATAAGGCGGTACCGTCCGAAATCAACTTCGGAAACGAAGTGGCACCGAGTCGGTGCT |
| **Fig. 1e. miR-17-5p guide** | CGAATTACCGGTCAACACTATGTGCTAACCTACCTACACTGTATGCACTTACTGTCCTCCGCGAATTCGTTTTAGAGCTAGAAAATAGCAAGTTAAAATAAGGCGGTACCGTCCGAAATCAACTTCGGAAACGAAGTGGCACCGAGTCGGTGCT |
| **Fig. 1e. miR-21-5p guide** | CGAATTACCGGTCAACACTGCAAATAAGATCAACTTCAGTCTAATAAGCACTGTCCTCCGCGAATTCGTTTTAGAGCTAGAAAATAGCAAGTTAAAATAAGGCGGTACCGTCCGAAATCAACTTCGGAAACGAAGTGGCACCGAGTCGGTGCT |
| **Fig. 1e. miR-122-5p guide** | CGAATTACCGGTCAACACTGTGTGTATCTCAAACTCCATTGTAACACTCACTGTCCTCCGCGAATTCGTTTTAGAGCTAGAAAATAGCAAGTTAAAATAAGGCGGTACCGTCCGAAATCAACTTCGGAAACGAAGTGGCACCGAGTCGGTGCT |
| **Fig. 1e. miR-206-3p guide** | CGAATTACCGGTCAACACTGTTTGTAAGGCCACAAACTTCCTAACATTCACTGTCCTCCGCGAATTCGTTTTAGAGCTAGAAAATAGCAAGTTAAAATAAGGCGGTACCGTCCGAAATCAACTTCGGAAACGAAGTGGCACCGAGTCGGTGCT |
| **Fig. 1f. miR-206-3p guide** | CGAATTACCGGTCAACACTGTTTGTAAGGCCACAAACTTCCTAACATTCACTGTCCTCCGCGAATTCGTTTTAGAGCTAGAAAATAGCAAGTTAAAATAAGGCGGTACCGTCCGAAATCAACTTCGGAAACGAAGTGGCACCGAGTCGGTGCT |
| **Fig. 1f miR-206-3p mimic sense** | TGGAATGTAAGGAAGTGTGTGG |
| **Fig. 1f miR 206-3p mimic antisense** | AACGCCATTATCACACTAAATA |
| **Fig. 1g miR-17-5p guide** | CGAATTACCGGTCAACACTATGTGCTAACCTACCTACACTGTATGCACTTACTGTCCTCCGCGAATTCGTTTTAGAGCTAGAAAATAGCAAGTTAAAATAAGGCGGTACCGTCCGAAATCAACTTCGGAAACGAAGTGGCACCGAGTCGGTGCT |
| **Fig. 1g miR-17-5p AntagomiR (IDT)** | CTACCTGCAGTGTAAGCACTTT (contains proprietary chemical modifications) |
| **Fig. 2b. miR guide (A)** | CGAATTACCGGTCAACACTATGTGCTAACCTACCTACACTGTATGCACTTACTGTCCTCCGCGAATTCGTTTTAGAGCTAGAAAATAGCAAGTTAAAATAAGGCGGTACCGTCCGAAATCAACTTCGGAAACGAAGTGGCACCGAGTCGGTGCT |
| **Fig. 2b. miR guide (B)** | CGAATTACCGGTCAACACTATGTGCTAACAGAGCTACCTACACTGTATGCACTTACTGTCCTCCGCGAATTCGTTTTAGAGCTAGAAAATAGCAAGTTAAAATAAGGCGGTACCGTCCGAAATCAACTTCGGAAACGAAGTGGCACCGAGTCGGTGCT |
| **Fig. 2b. miR guide (C)** | CGAATTACCGGTCAACACTATGTGCTAACAGAGTAGATAGGAACTACCTACACTGTATGCACTTACTGTCCTCCGCGAATTCGTTTTAGAGCTAGAAAATAGCAAGTTAAAATAAGGCGGTACCGTCCGAAATCAACTTCGGAAACGAAGTGGCACCGAGTCGGTGCT |
| **Fig. 2b. miR guide (D)** | CGAATTACCGGTCAACACTAAGTGCAAACAGAGTAGATAGGAACTACCTACACTGTATGCACTTACTGTCCTCCGCGAATTCGTTTTAGAGCTAGAAAATAGCAAGTTAAAATAAGGCGGTACCGTCCGAAATCAACTTCGGAAACGAAGTGGCACCGAGTCGGTGCT |
| **Fig. 2d. Blocker length 6 bp** | CGAATTACCGGTCAACACTATGTGCTAACCTACCTACACTGTATGCACTTACTGTCCTCCGCGAATTCGTTTTAGAGCTAGAAAATAGCAAGTTAAAATAAGGCGGTACCGTCCGAAATCAACTTCGGAAACGAAGTGGCACCGAGTCGGTGCT |
| **Fig. 2d. Blocker length 8 bp** | CGAATTCGAGGTCAACTCTATGTGCTAACCTACCTACACTGTATGCACTTACTGTCCTCCGCGAATTCGTTTTAGAGCTAGAAAATAGCAAGTTAAAATAAGGCGGTACCGTCCGAAATCAACTTCGGAAACGAAGTGGCACCGAGTCGGTGCT |
| **Fig. 2d. Blocker length 10 bp** | CGAATTCGCGATCAACTCTATGTGCTAACCTACCTACACTGTATGCACTTACTGTCCTCCGCGAATTCGTTTTAGAGCTAGAAAATAGCAAGTTAAAATAAGGCGGTACCGTCCGAAATCAACTTCGGAAACGAAGTGGCACCGAGTCGGTGCT |
| **Fig. 2f. miR guide (E)** | CGAATTACCGGTCAACACTATGTGCTAACCTACCTACACTGTATGCACTTACTGTCCTCCGCGAATTCGTTTTAGAGCTAGAAAATAGCAAGTTAAAATAAGGCGGTACCGTCCGAAATCAACTTCGGAAACGAAGTGGCACCGAGTCGGTGCT |
| **Fig. 2f. miR guide (F)** | CGAATTACCGGTCAACACTGTTTGTAAGGCCACAAACTTCCTAACATTCACTGTCCTCCGCGAATTCGTTTTAGAGCTAGAAAATAGCAAGTTAAAATAAGGCGGTACCGTCCGAAATCAACTTCGGAAACGAAGTGGCACCGAGTCGGTGCT |
| **Fig. 2f. miR guide (G)** | CGAATTACCGGTCAACACTGATTGTTAACCTACCTACACTGTAT***ACATTCA***CTGTCCTCCGCGAATTCGTTTTAGAGCTAGAAAATAGCAAGTTAAAATAAGGCGGTACCGTCCGAAATCAACTTCGGAAACGAAGTGGCACCGAGTCGGTGCT |
| **Fig. 2f. miR guide (H)** | CGAATTACCGGTCAACACTATGTGAAAGGCCACAAACTTCCTA***GCACTTA***CTGTCCTCCGCGAATTCGTTTTAGAGCTAGAAAATAGCAAGTTAAAATAAGGCGGTACCGTCCGAAATCAACTTCGGAAACGAAGTGGCACCGAGTCGGTGCT |
| **Fig. 3a. miR-17-5p Seed (2)** | CGAATAAAAGGTCAACACTATAAGCGAAAGCACTTACTGTCCTCCGCGAATTCGTTTTAGAGCTAGAAAATAGCAAGTTAAAATAAGGCGGTACCGTCCGAAATCAACTTCGGAAACGAAGTGGCACCGAGTCGGTGCT |
| **Fig. 3a. miR-16-5p Seed (2)** | CGAATTACCGGTCAACACTGATGCTGAAATGCTGCACTGTCCTCCGCGAATTCGTTTTAGAGCTAGAAAATAGCAAGTTAAAATAAGGCGGTACCGTCCGAAATCAACTTCGGAAACGAAGTGGCACCGAGTCGGTGCT |
| **Fig. 3a. miR-206-3p Seed (2)** | CGAATTACCGGTCAACACTGTTTGTGAAAACATTCACTGTCCTCCGCGAATTCGTTTTAGAGCTAGAAAATAGCAAGTTAAAATAAGGCGGTACCGTCCGAAATCAACTTCGGAAACGAAGTGGCACCGAGTCGGTGCT |
| **Fig. 3b. miR-17-5p Seed (2)** | CGAATAAAAGGTCAACACTATAAGCGAAAGCACTTACTGTCCTCCGCGAATTCGTTTTAGAGCTAGAAAATAGCAAGTTAAAATAAGGCGGTACCGTCCGAAATCAACTTCGGAAACGAAGTGGCACCGAGTCGGTGCT |
| **Fig. 3b. miR-17-5p Seed (3)** | CGAATAAAAGGTCAACACATTAAGCGAAAGCACTATCTGTCCTCCGCGAATTCGTTTTAGAGCTAGAAAATAGCAAGTTAAAATAAGGCGGTACCGTCCGAAATCAACTTCGGAAACGAAGTGGCACCGAGTCGGTGCT |
| **Fig. 3b. miR-17-5p Seed (4)** | CGAATAAAAGGTCAACACAATTAGCGAAAGCACATTCTGTCCTCCGCGAATTCGTTTTAGAGCTAGAAAATAGCAAGTTAAAATAAGGCGGTACCGTCCGAAATCAACTTCGGAAACGAAGTGGCACCGAGTCGGTGCT |
| **Fig. 3b. miR-17-5p Seed (5)** | CGAATAAAAGGTCAACACAATAAGCGAAAGCAATTTCTGTCCTCCGCGAATTCGTTTTAGAGCTAGAAAATAGCAAGTTAAAATAAGGCGGTACCGTCCGAAATCAACTTCGGAAACGAAGTGGCACCGAGTCGGTGCT |
| **Fig. 3b. miR-17-5p Seed (6)** | CGAATAAAAGGTCAACACAATATGCGAAAGCTCTTTCTGTCCTCCGCGAATTCGTTTTAGAGCTAGAAAATAGCAAGTTAAAATAAGGCGGTACCGTCCGAAATCAACTTCGGAAACGAAGTGGCACCGAGTCGGTGCT |
| **Fig. 3b. miR-17-5p Seed (7)** | CGAATAAAAGGTCAACACAATATTCGAAAGAACTTTCTGTCCTCCGCGAATTCGTTTTAGAGCTAGAAAATAGCAAGTTAAAATAAGGCGGTACCGTCCGAAATCAACTTCGGAAACGAAGTGGCACCGAGTCGGTGCT |
| **Fig. 3b. miR-17-5p Seed (8)** | CGAATAAAAGGTCAACACAATATGTGAAAACACTTTCTGTCCTCCGCGAATTCGTTTTAGAGCTAGAAAATAGCAAGTTAAAATAAGGCGGTACCGTCCGAAATCAACTTCGGAAACGAAGTGGCACCGAGTCGGTGCT |
| **Fig. 3c. crRNA WT** | CTGTCCTCCGCGAATTCGTTTCAGAGCTATGCTGGAAA |
| **Fig. 3c tracrRNA WT** | CAGCATAGCAAGTTGAAATAAGGCTAGTCCGTTATCAACTTGAAAAAGTGGCACCGAGTCGGTGCT |
| **Fig. 3c. crRNA miR-16-5p** | CGAATTACCGGTCAACACTGATGCTGAAATGCTGCACTGTCCTCCGCGAATTCGTTTCAGAGCTATGCTGGAAA |
| **Fig. 3c. crRNA miR-122-5p** | CGAATTACCGGTCAACACTGAATGTGAAAACACTCACTGTCCTCCGCGAATTCGTTTCAGAGCTATGCTGGAAA |
| **Fig. 3c. tracrRNA miR-17-5p** | GATTTCAACAAGCATAGCTATATAAGCGAAAGCACTTACAGCATAGCAAGTTGAAATAAGGCTAGTCCGTTATCAACTTGAAAAAGTGGCACCGAGTCGGTGCT |
| **Fig. 3c. tracrRNA miR-122-5p** | GATTTCAACAAGCATAGCTATGAATGTGAAAACACTCACAGCATAGCAAGTTGAAATAAGGCTAGTCCGTTATCAACTTGAAAAAGTGGCACCGAGTCGGTGCT |
| **Fig. 3c. miR-16-5p AntagomiR (IDT)** | CGCCAATATTTACGTGCTGCTA (contains proprietary chemical modifications) |
| **Fig. 3c. miR-17-5p AntagomiR (IDT)** | CTACCTGCAGTGTAAGCACTTT (contains proprietary chemical modifications) |
| **Fig. 4b-f. Standard guide (human)** | AAGAACACCTTCAGAACCGGGTTTCAGAGCTATGCTGGAAACAGCATAGCAAGTTGAAATAAGGCTAGTCCGTTATCAACTTGAAAAAGTGGCACCGAGTCGGTGCT |
| **Fig. 4b-f. Myo-miR guide (human)** | CCCGAAACTGTTGGTCATCTATGCTAGTGAAAACATTCAAAGAACACCTTCAGAACCGGGTTTCAGAGCTATGCTGGAAACAGCATAGCAAGTTGAAATAAGGCTAGTCCGTTATCAACTTGAAAAAGTGGCACCGAGTCGGTGCT |
| **Fig. 4b-f. Liver-miR guide (human)** | CCCGAAACTGTTGGTCATCTATGCTAGTGAAAACACTCAAAGAACACCTTCAGAACCGGGTTTCAGAGCTATGCTGGAAACAGCATAGCAAGTTGAAATAAGGCTAGTCCGTTATCAACTTGAAAAAGTGGCACCGAGTCGGTGCT |
| **Fig. 4g-j. Standard guide (mouse)** | AATTCAGATTCAGTGGGATGGTTTCAGAGCTATGCTGGAAACAGCATAGCAAGTTGAAATAAGGCTAGTCCGTTATCAACTTGAAAAAGTGGCACCGAGTCGGTGCT |
| **Fig. 4g-j. Myo-miR guide (mouse)** | CCATCCATCTATATCAAAATATGCTTGTGAAAACATTCAAATTCAGATTCAGTGGGATGGTTTCAGAGCTATGCTGGAAACAGCATAGCAAGTTGAAATAAGGCTAGTCCGTTATCAACTTGAAAAAGTGGCACCGAGTCGGTGCT |
| **Fig. 4g-j. Liver-miR guide (mouse)** | CCATCCATCTATATCAAAATATGATAGTGAAAACACTCAAATTCAGATTCAGTGGGATGGTTTCAGAGCTATGCTGGAAACAGCATAGCAAGTTGAAATAAGGCTAGTCCGTTATCAACTTGAAAAAGTGGCACCGAGTCGGTGCT |
| **Supp. Fig. 1c Dual-Luc miR-10a-5p** | TACCCTGTAGATCCGAATTTGTG |
| **Supp. Fig. 1c Dual-Luc miR-16-5p** | TAGCAGCACGTAAATATTGGCG |
| **Supp. Fig. 1c Dual-Luc miR-17-5p** | CAAAGTGCTTACAGTGCAGGTAG |
| **Supp. Fig. 1c Dual-Luc miR-21-5p** | TCAACATCAGTCTGATAAGCTA |
| **Supp. Fig. 1c Dual-Luc miR-122-5p** | TGGAGTGTGACAATGGTGTTTG |
| **Supp. Fig. 1c Dual-Luc miR-143-3p** | TGAGATGAAGCACTGTAGCTC |
| **Supp. Fig. 1c Dual-Luc miR-206-3p** | TGGAATGTAAGGAAGTGTGTGG |
| **Supp. Fig. 1d Dual-Luc miR-18a-5p** | TAAGGTGCATCTAGTGCAGATAG |
| **Supp. Fig. 1d Dual-Luc miR-20a-5p** | TAAAGTGCTTATAGTGCAGGTAG |
| **Supp. Fig. 1d Dual-Luc miR-106a-5p** | AAAAGTGCTTACAGTGCAGGTAG |
| **Supp. Fig. 1d Dual-Luc Let-7a-5p** | TGAGGTAGTAGGTTGTATAGTT |
| **Supp. Fig. 1e miR-10-5p guide scramble** | CGAATTACCGGTCGACTGAGACTAAAGAGCTTCACTAGTCCTCACTAGACTCTGTCCTCCGCGAATTCGTTTTAGAGCTAGAAAATAGCAAGTTAAAATAAGGCGGTACCGTCCGAAATCAACTTCGGAAACGAAGTGGCACCGAGTCGGTGCT |
| **Supp. Fig. 1e miR-16-5p guide scramble** | CGAATTACCGGTCAACACGCTAACAACGTTTACCTAATTGTCGCCGTAAGCCTGTCCTCCGCGAATTCGTTTTAGAGCTAGAAAATAGCAAGTTAAAATAAGGCGGTACCGTCCGAAATCAACTTCGGAAACGAAGTGGCACCGAGTCGGTGCT |
| **Supp. Fig. 1e miR-17-5p guide scramble** | CGAATTACCGGTCAACACTGGAATGAGTCGCAATTCAAACGTCTCTATCCTCTGTCCTCCGCGAATTCGTTTTAGAGCTAGAAAATAGCAAGTTAAAATAAGGCGGTACCGTCCGAAATCAACTTCGGAAACGAAGTGGCACCGAGTCGGTGCT |
| **Supp. Fig. 1e miR-122-5p guide scramble** | CGAATTACCGGTCAACACTGAGTAGATAGAACGTACCTCCTAACTACCCACTGTCCTCCGCGAATTCGTTTTAGAGCTAGAAAATAGCAAGTTAAAATAAGGCGGTACCGTCCGAAATCAACTTCGGAAACGAAGTGGCACCGAGTCGGTGCT |
| **Supp. Fig. 1e miR-206-3p guide scramble** | CGAATTACCGGTCAACACTTTGAGTAGTGTCCTACTCTCCACCACTCTAACTGTCCTCCGCGAATTCGTTTTAGAGCTAGAAAATAGCAAGTTAAAATAAGGCGGTACCGTCCGAAATCAACTTCGGAAACGAAGTGGCACCGAGTCGGTGCT |
| **Supp. Fig. 2b. miR-17-5p guide (2, 9, 17)** | CGAATTACCGGTCAACACTATGTGCTAACCTACCTACACTGTATGCACTTACTGTCCTCCGCGAATTCGTTTTAGAGCTAGAAAATAGCAAGTTAAAATAAGGCGGTACCGTCCGAAATCAACTTCGGAAACGAAGTGGCACCGAGTCGGTGCT |
| **Supp. Fig. 2b. miR-17-5p guide (2, 10-11)** | CGAATTACCGGTCAACACTATGTGCATTCCTACCTGCACTGATAGCACTTACTGTCCTCCGCGAATTCGTTTTAGAGCTAGAAAATAGCAAGTTAAAATAAGGCGGTACCGTCCGAAATCAACTTCGGAAACGAAGTGGCACCGAGTCGGTGCT |
| **Supp. Fig. 2b. miR-21-5p guide (2, 9, 17)** | CGAATTACCGGTCAACACTGCAAATAAGATCAACTTCAGTCTAATAAGCACTGTCCTCCGCGAATTCGTTTTAGAGCTAGAAAATAGCAAGTTAAAATAAGGCGGTACCGTCCGAAATCAACTTCGGAAACGAAGTGGCACCGAGTCGGTGCT |
| **Supp. Fig. 2b. miR-21-5p guide (2, 10-11)** | CGAATTACCGGTCAACACTGCAAATCATATCAACATCAGTAAGATAAGCACTGTCCTCCGCGAATTCGTTTTAGAGCTAGAAAATAGCAAGTTAAAATAAGGCGGTACCGTCCGAAATCAACTTCGGAAACGAAGTGGCACCGAGTCGGTGCT |
| **Supp. Fig. 3a miR-17-5p AntagomiR (IDT)** | CTACCTGCAGTGTAAGCACTTT (contains proprietary chemical modifications) |
| **Supp. Fig. 3a miR-17-5p (2) guide** | CGAATTACCGGTCAACACTATATGCGAAAGCACTTACTGTCCTCCGCGAATTCGTTTTAGAGCTAGAAAATAGCAAGTTAAAATAAGGCGGTACCGTCCGAAATCAACTTCGGAAACGAAGTGGCACCGAGTCGGTGCT |
| **Supp. Fig. 3a miR-17-5p guide (2, 9, 17)** | CGAATTACCGGTCAACACTATGTGCTAACCTACCTACACTGTATGCACTTACTGTCCTCCGCGAATTCGTTTTAGAGCTAGAAAATAGCAAGTTAAAATAAGGCGGTACCGTCCGAAATCAACTTCGGAAACGAAGTGGCACCGAGTCGGTGCT |
| **Supp. Fig. 3a miR-16-5p guide (2, 9, 17)** | CGAATTACCGGTCAACACTGATGCTAGTAGCCGCCATTATTTACATGCTGCACTGTCCTCCGCGAATTCGTTTTAGAGCTAGAAAATAGCAAGTTAAAATAAGGCGGTACCGTCCGAAATCAACTTCGGAAACGAAGTGGCACCGAGTCGGTGCT |
| **Supp. Fig. 4d**  **miR-17-5p seed** | CGAATTACCGGTCAACACTATATGCGAAAGCACTTACTGTCCTCCGCGAATTCGTTTTAGAGCTAGAAAATAGCAAGTTAAAATAAGGCGGTACCGTCCGAAATCAACTTCGGAAACGAAGTGGCACCGAGTCGGTGCT |
| **Supp. Fig. 5b-d**  **Alt. miR-17-5p guide** | CTACCTACACTGTATGCACTTAGTACTGTCCTCCGCGAATTCGTTTTAGAGCTAGCATACAGTGATAGCAAGTTAAAATAAGGCGGTACCGTCCGAAATCAACTTCGGAAACGAAGTGGCACCGAGTCGGTGCT |
| **Supp. Fig. 5b**  **Alt. miR-122-5p guide** | GCAAACTCCATTGTGACACTCAGTACTGTCCTCCGCGAATTCGTTTTAGAGCTAGTCACAATGGTATAGCAAGTTAAAATAAGGCGGTACCGTCCGAAATCAACTTCGGAAACGAAGTGGCACCGAGTCGGTGCT |
| **Supp. Fig. 5b**  **AntagomiR miR-17-5p** | CTACCTGCAGTGTAAGCACTTT (contains proprietary chemical modifications) |
| **Supp. Fig. 6a. miR-17-5p Seed (2)** | CGAATAAAAGGTCAACACTATAAGCGAAAGCACTTACTGTCCTCCGCGAATTCGTTTTAGAGCTAGAAAATAGCAAGTTAAAATAAGGCGGTACCGTCCGAAATCAACTTCGGAAACGAAGTGGCACCGAGTCGGTGCT |
| **Supp. Fig. 6a. Std. guide Mod1** | CTGTCCTCCGCGAATTCGTTTCAGAGCTAGAAAATAGCAAGTTAAAATAAGGCGGTACCGTCCGAAATCAACTTCGGAAACGAAGTGGCACCGAGTCGGTGCT |
| **Supp. Fig. 6a. miR-17-5p Seed (2) Mod1** | CGAATAAAAGGTCAACACTATAAGCGAAAGCACTTACTGTCCTCCGCGAATTCGTTTCAGAGCTAGAAAATAGCAAGTTAAAATAAGGCGGTACCGTCCGAAATCAACTTCGGAAACGAAGTGGCACCGAGTCGGTGCT |
| **Supp. Fig. 6a. Std. guide Optimized** | CTGTCCTCCGCGAATTCGTTTCAGAGCTATGCTGGAAACAGCATAGCAAGTTGAAATAAGGCTAGTCCGTTATCAACTTGAAAAAGTGGCACCGAGTCGGTGCT |
| **Supp. Fig. 6a. miR-17-5p Seed (2) Optimized** | CGAATAAAAGGTCAACACTATAAGCGAAAGCACTTACTGTCCTCCGCGAATTCGTTTCAGAGCTATGCTGGAAACAGCATAGCAAGTTGAAATAAGGCTAGTCCGTTATCAACTTGAAAAAGTGGCACCGAGTCGGTGCT |
| **Supp. Fig. 6b. miR-17-5p Seed (2) Optimized** | CGAATTCAAGGTCAACACTATAAGCGAAAGCACTTACTGTCCTCCGCGAATTCGTTTCAGAGCTATGCTGGAAACAGCATAGCAAGTTGAAATAAGGCTAGTCCGTTATCAACTTGAAAAAGTGGCACCGAGTCGGTGCT |
| **Supp. Fig. 6b. miR-18a-5p Seed (2) Optimized** | CGAATTCAAGGTCAACACTAGAAGCGAAAGCACCTACTGTCCTCCGCGAATTCGTTTCAGAGCTATGCTGGAAACAGCATAGCAAGTTGAAATAAGGCTAGTCCGTTATCAACTTGAAAAAGTGGCACCGAGTCGGTGCT |
| **Supp. Fig. 6b. Let-7a-5p Seed (2) Optimized** | CGAATTCAAGGTCAACACAAGATAGGAAACTACCTACTGTCCTCCGCGAATTCGTTTCAGAGCTATGCTGGAAACAGCATAGCAAGTTGAAATAAGGCTAGTCCGTTATCAACTTGAAAAAGTGGCACCGAGTCGGTGCT |
| **Supp. Fig. 6c. miR-17-5p Seed (2) Optimized** | CGAATTCAAGGTCAACACTATAAGCGAAAGCACTTACTGTCCTCCGCGAATTCGTTTCAGAGCTATGCTGGAAACAGCATAGCAAGTTGAAATAAGGCTAGTCCGTTATCAACTTGAAAAAGTGGCACCGAGTCGGTGCT |
| **Supp. Fig. 6c. miR-18a-5p Seed (2) Optimized** | CGAATTCAAGGTCAACACTAGAAGCGAAAGCACCTACTGTCCTCCGCGAATTCGTTTCAGAGCTATGCTGGAAACAGCATAGCAAGTTGAAATAAGGCTAGTCCGTTATCAACTTGAAAAAGTGGCACCGAGTCGGTGCT |
| **Supp. Fig. 6c. miR-20a-5p Seed (2) Optimized** | CGAATTCAAGGTCAACACAAAGAGCGAAAGCACTTACTGTCCTCCGCGAATTCGTTTCAGAGCTATGCTGGAAACAGCATAGCAAGTTGAAATAAGGCTAGTCCGTTATCAACTTGAAAAAGTGGCACCGAGTCGGTGCT |
| **Supp. Fig. 6c. miR-106a-5p Seed (2) Optimized** | CGAATTCAAGGTCAACACTAAGAGCGAAAGCACTTACTGTCCTCCGCGAATTCGTTTCAGAGCTATGCTGGAAACAGCATAGCAAGTTGAAATAAGGCTAGTCCGTTATCAACTTGAAAAAGTGGCACCGAGTCGGTGCT |
| **Supp. Fig. 6c. Let-7a-5p Seed (2) Optimized** | CGAATTCAAGGTCAACACAAGATAGGAAACTACCTACTGTCCTCCGCGAATTCGTTTCAGAGCTATGCTGGAAACAGCATAGCAAGTTGAAATAAGGCTAGTCCGTTATCAACTTGAAAAAGTGGCACCGAGTCGGTGCT |
| **Supp. Fig. 6c. miR-17-5p AntagomiR (IDT)** | CTACCTGCAGTGTAAGCACTTT (contains proprietary chemical modifications) |
| **Supp. Fig. 6c. miR-18a-5p AntagomiR (IDT)** | CTATCTGCACTAGATGCACCTTA (contains proprietary chemical modifications) |
| **Supp. Fig. 6c. miR-20a-5p AntagomiR (IDT)** | CTACCTGCACTATAAGCACTTTA (contains proprietary chemical modifications) |
| **Supp. Fig. 6c. miR-106a-5p AntagomiR (IDT)** | CTACCTGCACTGTAAGCACTTTT (contains proprietary chemical modifications) |
| **Supp. Fig. 6c. Let-7a-5p AntagomiR (IDT)** | AACTATACAACCTACTACCTCA (contains proprietary chemical modifications) |
| **Supp. Fig. 6d. miR-18a-5p Seed (2) Optimized** | CGAATTCAAGGTCAACACTAGAAGCGAAAGCACCTACTGTCCTCCGCGAATTCGTTTCAGAGCTATGCTGGAAACAGCATAGCAAGTTGAAATAAGGCTAGTCCGTTATCAACTTGAAAAAGTGGCACCGAGTCGGTGCT |
| **Supp. Fig. 6c. miR-18a-5p AntagomiR (IDT)** | CTATCTGCACTAGATGCACCTTA (contains proprietary chemical modifications) |
| **Supp. Fig. 6g. miR-17-5p guide (2, 9, 17)** | CGAATTACCGGTCAACACTATGTGCTAACCTACCTACACTGTATGCACTTACTGTCCTCCGCGAATTCGTTTTAGAGCTAGAAAATAGCAAGTTAAAATAAGGCGGTACCGTCCGAAATCAACTTCGGAAACGAAGTGGCACCGAGTCGGTGCT |
