# Supplementary material for: Tissue-specific modulation of CRISPR activity by miRNA-sensing guide RNAs
Source: Nucleic Acids Res. 2025 Jan 22;53(2):gkaf016. doi: 10.1093/nar/gkaf016 (PMC11754125; doi:10.1093/nar/gkaf016)
Supplement: gkaf016_Supplemental_Files [file gkaf016_supplemental_files.zip › Supplementary Table 2.docx]

**SUPPLEMENTARY TABLE 2**

**TaqMan assays used for miRNA quantification**

| Target | Species | Product ID | Detection channel |
| --- | --- | --- | --- |
| miR-10a-5p | human | 479241_mir | FAM |
| miR-16-5p | human | 477860_mir | FAM |
| miR-17-5p | human | 478447_mir | FAM |
| miR-21-5p | human | 477975_mir | FAM |
| miR-122-5p | human | 477855_mir | FAM |
| miR-143-3p | human | 477912_mir | FAM |
| miR-206-3p | human | 477968_mir | FAM |

**Primer sequences**

| ID | Sequence (5ʹ to 3ʹ) |
| --- | --- |
| Supp. Fig. 3 hATG2B_F | CTTCAGATGGAGTTGGAGGAGAC |
| Supp. Fig. 3 hATG2B_R | AGTGGCTCCTTTCAGTCCTACG |
| Supp. Fig. 3 hNUP35_F | CCCAGGACTTGGATCAACACCT |
| Supp. Fig. 3 hNUP35_R | CGTCTTTCGTGGCTGACCGATA |
| Supp. Fig. 3 hTMEM127_F | CTCTCCGCTTTCCTTCTGGATG |
| Supp. Fig. 3 hTMEM127_R | AATGACGGTGGCACACTGCAGA |
| Supp. Fig. 3 hHPRT_F | CATTATGCTGAGGATTTGGAAAGG |
| Supp. Fig. 3 hHPRT_R | CTTGAGCACACAGAGGGCTACA |
| Fig. 4b. hsa-DMD-Ex51_F | GAAACTGCCATCTCCAAACTAGAAA |
| Fig. 4b. hsa-DMD-Ex54_R | TCATGTGGACTTTTCTGGTATCATC |
