# Supplementary material for: Tissue-specific modulation of CRISPR activity by miRNA-sensing guide RNAs
Source: Nucleic Acids Res. 2025 Jan 22;53(2):gkaf016. doi: 10.1093/nar/gkaf016 (PMC11754125; doi:10.1093/nar/gkaf016)
Supplement: gkaf016_Supplemental_Files [file gkaf016_supplemental_files.zip › Supplementary Table 3.docx]

**SUPPLEMENTARY TABLE 3**

**Antibodies used for protein detection**

| **WesternBlot** |  |  |  |  |
| --- | --- | --- | --- | --- |
|  | Manufacturer | reference | Source | Dilution |
| Dystrophin | Leica | NCL-DYS1 | Mouse | 1:100 |
| Vinculin | Sigma | V9131 | Mouse | 1:200 |
| Anti-mouse HRP | CellSignal | 7076 | Horse | 1:5,000 |
| Anti-Rabbit 800 | Abcam | ab216773 | Goat | 1:10,000 |
|  |  |  |  |  |
| **IHC** |  |  |  |  |
|  | Manufacturer | reference | Source | Dilution |
| Dystrophin | Abcam | 15277 | Rabbit | 1:1,000 |
| Laminin α-2 chain | Sigma | L0663 | Rat | 1:1,000 |
| Anti-Rabbit Alexa 594 | Abcam | ab150080 | Goat | 1:500 |
| Anti-Rat Alexa 488 | Abcam | ab150157 | Goat | 1:500 |
|  |  |  |  |  |
| **ICC** |  |  |  |  |
|  | Manufacturer | reference | Source | Dilution |
| Dystrophin | Abcam | 15277 | Rabbit | 1:400 |
| Vinculin | Sigma | V9131 | Mouse | 1:400 |
| Anti-Rabbit Alexa 594 | Abcam | ab150080 | Goat | 1:500 |
| Anti-Mouse Alexa 488 | Abcam | ab150113 | Goat | 1:500 |
